# Reference Values for Postmortem Examination of the Heart in the Macropod (*Macropodidae*) and Koala (*Phascolarctidae*)

**DOI:** 10.3390/ani15101397

**Published:** 2025-05-12

**Authors:** Ella Cousins, Lucy Woolford, David McLelland, Sarah Brownrigg, Natasha Speight

**Affiliations:** 1 School of Animal and Veterinary Sciences, Faculty of Sciences, Engineering and Technology, University of Adelaide, Roseworthy, SA 5371, Australia; 2 Zoos South Australia, Frome Rd., Adelaide, SA 5000, Australia

**Keywords:** cardiovascular disease, diagnosis, kangaroo, morphology, morphometric, Phascolarctos

## Abstract

Morphometric cardiac reference values are reported for macropods and koalas (*Phascolarctos cinereus*). Body weight (BW), heart weight (HW), left ventricle (LV) wall, interventricular septum (S), right ventricle (RV) wall thickness, and LV+S and RV weights were measured at postmortem examination of 48 macropods and 32 koalas that had no evidence of cardiovascular disease. The HW/BW% (0.43–0.96%) and (LV+S)/RV (2.80–4.22) for macropods were comparable to domestic species. In koalas, the HW/BW% (0.25–0.51%) was lower, and the (LV+S)/RV (3.06–5.41) ranged higher than in macropods and domestic species. The LV:RV of koalas (1.0–10.8) was more variable than in macropods (1.17–4.27). Two macropods with cardiac disease were assessed on postmortem examination against the generated reference values. An adult male common wallaroo (*Osphranter robustus*) was found dead with copious serous peritoneal effusion, chronic passive hepatic congestion with centrilobular fibrosis, and dilation of the RV, while the LV:RV was elevated, supportive of RV thinning. A 21-year-old female zoo-housed Matschie’s tree kangaroo (*Dendrolagus matschiei*) had a flaccid thin-walled RV, LV cardiomyocyte hypertrophy, interstitial myocardial fibrosis and myofiber degeneration, pulmonary oedema, and serous pericardial effusion. The (LV+S)/RV and LV:RV were elevated and RV:S decreased, supporting left hypertrophic cardiomyopathy. Species-specific reference values presented in this study facilitate objective and improved postmortem cardiac assessment in macropods and koalas.

## Simple Summary

Morphological assessment of the heart is an integral component of postmortem investigation of sudden death and cardiac disease in domestic animals and wildlife species. Reference values for these parameters have been published for domestic species, however, no postmortem reference values for these morphometrics are available for Australian marsupials, such as macropods (Macropodidae: kangaroos, wallabies, tree kangaroos, and others) and koalas (*Phascolarctos cinereus*). Species-specific reference values presented in this study facilitate objective and improved postmortem cardiac assessment in macropods and koalas.

## 1. Introduction

Establishing health status and diagnosing disease can be challenging in native Australian marsupials, such as macropods (Macropodidae: kangaroos, wallabies, tree kangaroos, and others) and koalas (*Phascolarctos cinereus*). This is due to a lack of species-specific parameters available to assess their often unique morphological characteristics. Given that some macropod species, and koalas, have an IUCN classification of ‘endangered’, due to threats such as habitat loss, motor vehicle accident, and disease [1,2,3], it is important to build upon our foundations of understanding of these parameters and their applications in wildlife veterinary medicine.

Morphological assessment of the heart is an integral component of postmortem investigation of sudden death and cardiac disease in domestic animals and wildlife species [4]. This involves macroscopic visual assessment for pathologic lesions, morphometric measurements, and histopathological evaluation of the endo-, myo-, and epicardium and the conduction system. Morphometric measurements include total heart weight, ventricular wall weights, and ventricular wall thicknesses [4,5]. Reference values for these parameters have been published for domestic species, including the dog, horse, cat, cow, goat, sheep, and pig [4]. However, no postmortem reference values for these morphometrics are available for Australian marsupials, such as macropods and koalas, recently highlighted as a significant obstacle in the investigation of cardiac disease in these species [6].

Macropods and koalas from the wild and those managed in human care internationally in zoological institutions are often the subjects of pathological investigations. The lack of reference values for cardiac morphometrics makes the diagnosis of cardiac disease, and specifically cardiomyopathies, i.e., those with absence of significance congenital or acquired valvular or vascular abnormality, or extrinsic or secondary (known systemic or etiological abnormalities) contributing factors, in these species difficult. Few studies describe normal cardiac anatomy and physiology in marsupials [7,8]. One study in wallabies and kangaroos suggested that heart rate, arterial pressure, and cardiac output were similar to those of domestic species of comparable size [9]. Detailed descriptions of the cardiovascular anatomy and physiology of the koala are lacking in the literature, with small numbers of published reports comparing koala anatomy with eutherian mammals [10,11,12].

In macropods, cardiac disease is most reported in association with nutritional myopathies, exertional myopathies, and infectious disease (e.g., encephalomyocarditis virus, *Toxoplasma gondii*, and *Neosporum caninum*) [13,14,15]. Reports of cardiomyopathies in marsupials are limited to individual case reports, small comparative studies, and zoological collection surveys, with morphometric assessment of the heart often limited or not performed at all. Hypertrophic cardiomyopathy has been diagnosed in a three-year-old, intact male Matschie’s tree kangaroo (*Dendrolagus matschiei*) [16] and in two captive Bennett’s wallabies (*Macropus rufogriseus rufogriseus*) [17]. Right-sided cardiac hypertrophy attributed to hypoxia of high altitude has been described in an adult male Bennett’s wallaby held in a zoological collection situated at an altitude above 2000 m [18]. A degenerative cardiomyopathy of unspecified aetiology was reported in the eastern grey kangaroo (*Macropus giganteus*) [19]. More broadly, cardiovascular lesions suggestive of hypertension were identified in multiple western grey kangaroos from a single zoo. No aetiology was determined; however, lesions were characterised by arterial medial thickening and hypertrophy and increased tortuosity of renal arterioles. In koalas, cardiac failure has been reported secondary to an atrial septal defect [20]. The major challenge for clinicians and pathologists investigating cardiomyopathies, and cardiac disease more generally, in marsupials at postmortem is the lack of species-specific cardiac morphometric reference values. This often leads to a reliance on comparing individuals with conspecifics, which are generally few, if indeed available.

The aim of this study was to develop cardiac morphometric reference values for koalas and commonly examined macropod species to improve pathological assessment of the heart in these taxa.

## 2. Materials and Methods

Forty-eight macropods that had died of causes unrelated to cardiovascular disease were opportunistically sampled during routine postmortem examination, including four captive zoo-based wallabies and kangaroos that died or were euthanised due to health concerns, twenty rescued free-ranging kangaroos and wallabies with traumatic injuries necessitating euthanasia at referring veterinary clinics or zoos, and twenty-four free-ranging kangaroos that were euthanised under a permit issued by the South Australian Department of Environment of Water in accordance with the Animal Welfare Act 1985, for purposes not related to this study. By species, the study population comprised 12 red kangaroos (*Osphranter rufus*), 24 western grey kangaroos (*M. fuliginosus*), 8 tammar wallabies (*Notamacropus eugenii*), and 4 yellow-footed rock wallabies (*Petrogale xanthopus*); by sex, there were 29 females and 19 males (Table 1).

A total of 32 koalas, free ranging from the Mount Lofty Ranges region of South Australia (SA) or housed at Adelaide Zoo, SA, which had died of causes unrelated to cardiovascular disease, were obtained opportunistically for routine postmortem examination. Of these, 27 were euthanised on welfare grounds due to a dog attack or other trauma, and 5 had died due to other non-cardiovascular-related disease. Nineteen koalas were female and thirteen were male (Table 1). Of the 32 koalas, 31 had approximate age determined by tooth wear of the upper premolar, allowing evaluation by tooth wear class (TWC; I–V) [11]: TWC I, *n* = 2; class II, *n* = 3; class III, *n* = 12; class IV, *n* = 11; class V, *n* = 3.

Additionally, an adult male free-ranging common wallaroo (*Osphranter robustus*) (Case 1) found dead within the boundaries of an open-range zoo, and an adult female zoo-housed Matschie’s tree kangaroo (Case 2) euthanised on welfare grounds due to deteriorating degenerative joint disease, were presented for postmortem examination. Both were found to have gross lesions suggestive of cardiac disease, and data were collected for opportunistic evaluation against the reference values generated here.

**Figure 1:**
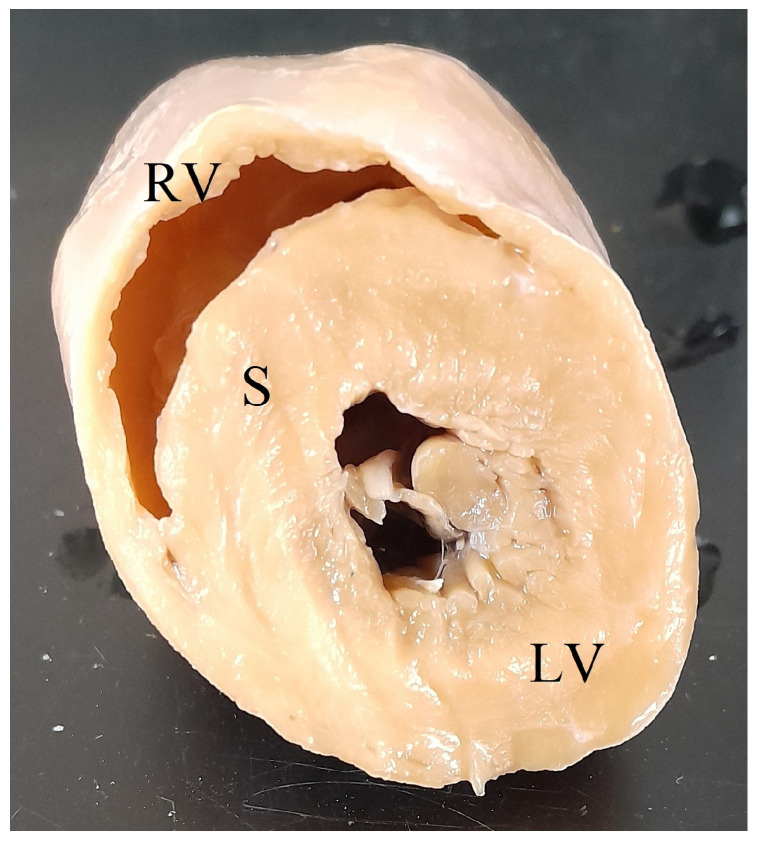
Transected koala heart showing right ventricular free wall (RV), interventricular septum (S), and left ventricular free wall (LV).

Following routine postmortem examination, the heart was transected approximately one-third the length of the ventricles above the apex, cleared of blood, weighed in toto, and fixed in 10% neutral buffered formalin. Left ventricular free wall (LV), interventricular septum (S), and right ventricular free wall (RV) widths were measured at the narrowest point of the free wall or septum at the site of transection (Figure 1). LV+S weight and RV weight were determined as per Robinson and Robinson, 2016 [4]. Minimum histopathology tissue sets included the heart, liver, and lung to screen for microscopic cardiac lesions and systemic lesions associated with cardiac insufficiency. A complete set of tissues was collected for histopathology for cases where the cause of death was not evident grossly. Formalin-fixed tissue samples were processed by routine histopathological techniques, embedded in paraffin, sectioned at 4 µm, and stained with haematoxylin and eosin (HE). Tissues were examined by using an Olympus BX43 microscope (Olympus, Tokyo, Japan).

Mean, standard deviation (SD), and maximum and minimum values of HW/BW (%), (LV+S)/RV, LV:RV, LV:S, RV:S, (LV+S)/BW (%), and (LV+S)/HW (%) were determined for koalas, for all macropods, for kangaroos (*Osphranter* spp. and *Macropus* spp.), and for wallabies (*Notamacropus* spp. and *Petrogale* spp.) in Excel (Microsoft Corporation, Redmond, WA, USA, 2021). For 3/48 macropods and 26/32 koalas, heart weight and total body weight were not collected. All animals in the study populations were determined to be unaffected by cardiovascular disease. Data were also examined using Reference Value Advisor (National Veterinary School of Toulouse, Toulouse, Haute-Garonne, France; version 2.1) within Excel. Histograms were inspected visually for normality and detection of outliers. A tendency to retain rather than remove outlier values was adopted, unless there were determinable clinical or data integrity reasons for removal. No outliers were removed. For sex comparison, descriptive statistics were generated, and data were examined for normality using the Shapiro–Wilk test in SPSS v29 (IBM, New York, NY, USA). No outliers were removed. Non-parametric data were analysed using the Mann–Whitney U test, with statistical significance regarded as *p* < 0.05.

## 3. Results

### 3.1. Morphometric Reference Values

Heart weight as a percentage of total body weight for 45 macropods and 6 koalas is presented in Table 2. The ratios of the left ventricular free wall and septum weight to right ventricular free wall weight ((LV+S)/RV) are presented in Table 3. Ventricular wall width ratios (LV:RV, LV:S, and RV:S) for 48 macropods and 32 koalas are presented in Table 4, Table 5 and Table 6. Broad reference values for LV:RV were observed for both the macropod (1.17–4.27) and the koala (1.00–10.84), with koala values notably wider than the macropod. Additional values generated included LV+S weight as a percentage of total body weight (BW; Table 7) and as a percentage of total heart weight (HW; Table 8).

For kangaroos, females showed a significantly higher HW/BW% than males (0.73% ± 0.03 vs. 0.63% ± 0.02; *p* = 0.018). Also, female koalas (*n* = 13) showed a significantly higher LV:RV width ratio (6.9 ± 0.7) than males (*n* = 19; 5.3 ± 0.6; *p* = 0.037) (Supplementary file S1).

### 3.2. Pathologic Cases

**Figure 2:**
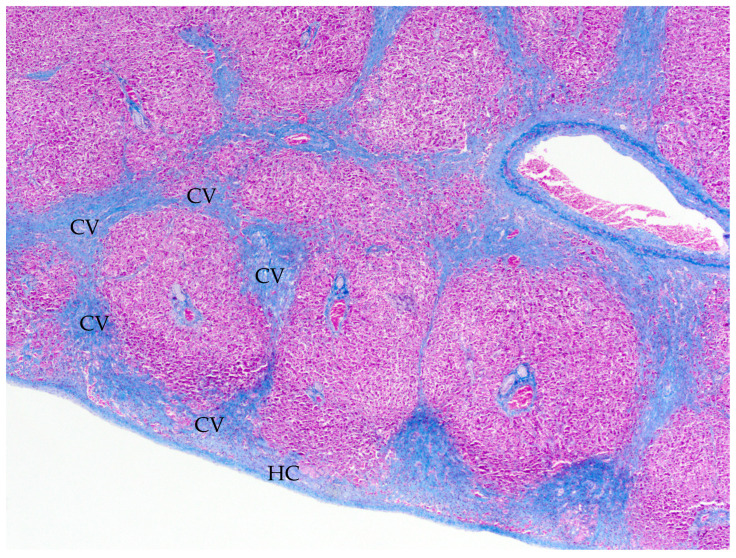
Case 1, common wallaroo (*O. robustus*), liver, Masson’s Trichrome stain. There is marked expansion of fibrocollagenous connective tissue around central veins (CVs) with centrilobular bridging fibrosis and fibrous thickening of the hepatic capsule (HC).

Cardiac measurements for two macropods suspected to have cardiac disease were compared with the reference values established in this study (Table 9). Case 1 was an adult male free-ranging common wallaroo (*Osphranter robustus*) that was found dead. The kangaroo was moderately underconditioned; however, body weight was not recorded. At postmortem examination, there was copious yellow serous fluid in the peritoneal cavity. The right ventricle was subjectively distended. For ventricular widths, LV:RV was elevated, supporting RV thinning, and LV:S was within reference values for all macropods but marginally elevated for kangaroos. Other available measurements were within the reference values. Microscopic examination of the left and right myocardium was unremarkable on HE and Masson’s Trichrome stains. The liver was congested with marked chronic centrilobular and bridging fibrosis and subcapsular fibrosis with subcapsular congestion and hepatocellular atrophy, consistent with chronic passive congestion (Figure 2). Occasional aberrant ductular profiles were adjacent centrilobular fibrosis.

**Figure 3:**
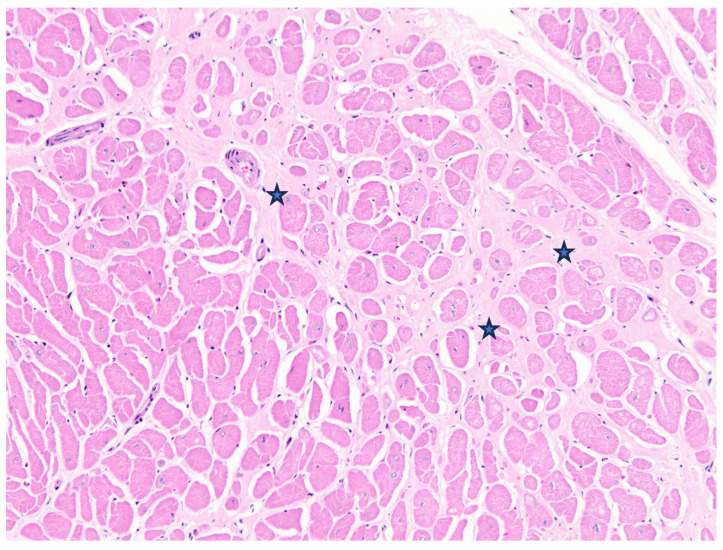
Case 2, Matschie’s tree kangaroo (*D. matschiei*), left ventricular myocardium, haematoxylin and eosin stain. Cardiomyocytes are hypertrophic with increased variation in size. Mature fibrocollagenous connective tissue dissects between and isolates individual fibres (stars).

Case 2 was a 21-year-old female zoo-housed Matschie’s tree kangaroo weighing 8.69 kg. This geriatric kangaroo was euthanised due to deterioration of chronic vertebral osteoarthritis that had been managed with non-steroidal anti-inflammatory medication for the previous three years. At postmortem examination, the right ventricle was subjectively thin-walled and flaccid. The lungs oozed serous fluid on the cut section, and there was serosanguinous fluid in the large airways and pericardial sac. The (LV+S)/RV was elevated above reference values; however, HW/BW% was within the reference values. For ventricular wall ratios, LV:RV was elevated and RV:S decreased, suggesting left-side ventricular hypertrophy and/or thinning of the RV wall. Microscopically, there was moderate cardiomyocyte hypertrophy in the left ventricle, with multifocal interstitial myocardial fibrosis, myofiber degeneration, loss, and atrophy, with interstitial oedema (Figure 3). The lungs were diffusely congested, airways often flooded by proteinaceous fluid, with diapedesis and increased foamy macrophages, and haemosiderophages were not observed.

## 4. Discussion

Species-specific reference values facilitate objective assessment of the heart at postmortem. Determined reference values (mean ± 2SD) for heart weight as a percentage of body weight in macropods were similar to those determined previously for the tammar wallaby [21] and comparable to the domestic dog and horse. In contrast, koala reference values were lower with a narrower range than for macropods, dogs, and horses, but comparable to published reference values for the pig [1]. In the kangaroo, HW/BW% was greater in female than male kangaroos, contrasting to most domestic animals in which HW/BW% is typically greater in males than in females [4]. This most likely reflects sexual dimorphism in body size, with male red kangaroos and western grey kangaroos weighing 22–92 kg and 18–72 kg, respectively, compared to their female counterparts, with females of both species weighing between 17 kg and 39 kg [22,23].

The heart weight as a % of body weight is higher in more athletic species (horses and dogs) and is increased by training and exercise [4]. Kangaroos are athletic species, able to cover ground in short bursts at high speed [24]. Movements of >100 km have been recorded for individual red kangaroos from tagging and radio telemetry [25,26]. However, outside of drought periods when animals range more widely [27], these have been considered exceptions in a generally sedentary population where individuals move within home ranges of variable size [28]. In the koala, the lower heart weight as a % of body weight may reflect the relatively sedentary nature of these animals, which have been found to have a low metabolic rate, 74% of the predicted marsupial value [29].

The ratio of the left ventricle and septum weight to the right ventricular weight ((LV+S)/RV) is a key parameter used to assess the heart in domestic animals [4]. A ratio between 2.8 and 4.0 is considered normal in mature domestic animals, with a ratio > 4 indicating left ventricular hypertrophy, and a ratio < 2.8 suggesting right ventricular hypertrophy. For macropods, the reference values were comparable with domestic species; however, the koala values were broader, with a higher upper reference value.

Assessment of ventricular wall ratios is commonplace in postmortem assessment of the heart: in comparison to the domestic dog (1.80–2.46), a broader range of values was observed for LV:RV in both the macropod (1.165–4.27) and, notably, the koala (1.00–10.84), with the latter species showing an upper reference value and maximum twice that of macropods. It is possible that the broad reference values for LV:RV in the koala make this a less sensitive measure for the investigation of cardiac disease than in other species.

In Case 2, elevated (LV+S)/RV and LV:RV, and decreased RV:S, provided supportive evidence for left ventricular hypertrophy and left-sided congestive heart failure, alongside corroborative findings of myocardial hypertrophy with myofiber degeneration, and interstitial fibrosis, pulmonary oedema, and serous pericardial effusion. These findings were consistent with expected lesions in hypertrophic cardiomyopathy of felids and other domestic species [30]; however, HW/BW% was not increased in Case 2. For Case 1, elevated LV:RV lent further weight to grossly observed RV thinning and probable right-sided congestive heart failure, supported by chronic passive congestive and fibrotic changes in the liver and ascites observed grossly. Histopathological findings in the liver mirrored expected lesions in domestic animals with chronic right-sided congestive heart failure [30,31]. Hypertrophic cardiomyopathy has been previously described in two captive Bennett’s wallabies [17]. Although body and heart weights were not published, reported (LV+S)/RV values of 5 and 8.2, for each wallaby, respectively, were elevated above the reference values determined in this study, lending further support for HCM in these individuals. Conversely, LV:RV, LV:S, and RV:S were within the RI generated here. Other reports of cardiomyopathies in macropods reviewed during this study did not include sufficient information to allow assessment of hearts against generated reference values. Future studies will benefit from larger sample sizes and expansion of the number of species examined.

## 5. Conclusions

Reference values developed in this study facilitated improved and objective postmortem cardiac assessment in macropods and koalas. As variation in heart morphology is expected across domestic species, it is likely that there is variation across the Macropodidae. This large superfamily includes kangaroos, wallabies, and the musky rat kangaroo (*Hypsiprymnodon moschatu*), which exhibit diversity in size, anatomy, and life histories [32]. Overall, however, these parameters will allow a better understanding of the occurrence and characteristics of cardiac disease in macropods and koalas.

## Figures and Tables

**Table 1:** Reference population for determination of macropod and koala cardiac morphometric intervals. TWC = tooth wear class.

		Western Grey Kangaroo (24)	Red Kangaroo (12)	Tammar Wallaby (8)	Yellow-Footed Rock Wallaby (4)	Koala (32)
Sex	Male	11	6	2	0	13
	Female	13	6	6	4	19
Age	Juvenile (<2 years)	9	2	3	0	TWC I = 2
	Adult (>2 years)	15	10	5	4	TWC II = 3 TWC III = 12 TWC I = 11 TWC V= 3

**Table 2:** Heart weight as a percentage of body weight for healthy macropods and koalas, within inclusion of published intervals for domestic species. YFRW = yellow-footed rock wallaby.

Species (*n*)	Mean (%)	Mean ± 2SD (%)	Min. (%)	Max. (%)
All Macropods (45)	0.69	0.43–0.96	0.50	1.13
Kangaroos—Red, Western grey (34)	0.68	0.43–0.93	0.50	1.13
Wallabies—YFRW, Tammar (11)	0.73	0.42–1.03	0.60	1.06
Tammar Wallaby (6) [14]	0.67	0.51–0.83	0.53	0.73
Koala (6)	0.38	0.25–0.51	0.31	0.49
Dog (21) [1]	0.71	0.43–0.99	n/a	n/a
Dog [2]	0.76	0.58–0.94	n/a	n/a
Horse (12) [1]	0.69	0.41–0.97	n/a	n/a
Cat (9) [1]	0.58	0.28–0.88	n/a	n/a
Cow (15) [1]	0.48	0.30–0.66	n/a	n/a
Goat (11) [1]	0.46	0.26–0.66	n/a	n/a
Sheep (8) [1]	0.41	0.17–0.65	n/a	n/a
Pig (8) [1]	0.40	0.32–0.48	n/a	n/a

**Table 3:** Left ventricular free wall and septum to right ventricular free wall weight ratios ((LV+S)/RV) for healthy macropods and koalas compared to published intervals for domestic species.

Species (*n*)	Mean	Mean ± 2SD	Min.	Max.
All Macropods (48)	3.52	2.80–4.22	2.68	4.28
Kangaroos—Red, Western grey (36)	3.42	2.80–4.03	2.68	4.04
Wallabies—YFRW, Tammar (12)	3.81	3.16–4.46	3.39	4.28
Koala (6)	4.23	3.06–5.41	3.77	5.29
Dog (21) [1]	3.26	2.39–5.12	n/a	n/a
Dog [2]	3.32	2.76–3.88	n/a	n/a
Horse (12) [1]	3.12	2.43–4.34	n/a	n/a
Cat (9) [1]	3.45	2.94–4.17	n/a	n/a
Cow (15) [1]	2.78	2.43–4.00	n/a	n/a
Goat (11) [1]	3.12	2.50–4.17	n/a	n/a
Sheep (8) [1]	3.33	2.63–4.45	n/a	n/a
Pig (8) [1]	2.94	2.38–3.84	n/a	n/a

**Table 4:** Left-to-right ventricular wall width ratios (LV:RV) determined at postmortem for healthy macropods, koalas, and dogs.

Species (*n*)	Mean	Mean ± 2SD	Min.	Max.
All Macropods	2.70 (48)	1.15–4.26	1.60	4.67
Kangaroos (Red, Western Grey)	2.51 (36)	1.20–3.78	1.60	4.00
Wallabies (YRFW, Tammar)	3.35 (12)	1.74–4.97	2.00	4.67
Koala	5.92 (32)	1.00–10.84	2.75	11.00
Dog [2]	2.13	1.80–2.46	n/a	n/a

**Table 5:** Left ventricular wall to septum width ratios for healthy macropods and koalas.

Species (*n*)	Mean	Mean ± 2SD	Min.	Max.
All Macropods (48)	1.08	0.77–1.40	0.83	1.67
Kangaroos—Red, Western Grey (36)	1.06	0.78–1.35	0.83	1.67
Wallabies—YFRW, Tammar (12)	1.14	0.77–1.51	0.89	1.50
Koala (32)	1.08	0.61–1.54	0.82	1.83

**Table 6:** Right ventricular wall to septum width ratios for healthy macropods and koalas.

Species (*n*)	Mean	Mean ± 2SD	Min.	Max.
All Macropods (48)	0.42	0.23–0.61	0.21	0.67
Kangaroos—Red, Western Grey (36)	0.44	0.27–0.62	0.29	0.67
Wallabies—YFRW, Tammar (12)	0.35	0.19–0.52	0.21	0.56
Koala (32)	0.21	0.00–0.43	0.09	0.67

**Table 7:** Left ventricle and septum weight as a percentage of body weight, (LV+S)/BW (%), for healthy macropods and koalas.

Species (*n*)	Mean (LV+S)/BW (%)	Mean ± 2SD	Min.	Max.
All Macropods (45)	0.43	0.25–0.60	0.30	0.74
Kangaroos—Red Western Grey (34)	0.42	0.25–0.59	0.30	0.74
Wallabies—YFRW, Tammar (11)	0.44	0.24–0.64	0.32	0.68
Koala (6)	0.22	0.16–0.29	0.19	0.22

**Table 8:** Left ventricle and septum weight as a percentage of heart weight, (LV+S)/HW (%), for healthy macropods and koalas.

Species (*n*)	Mean (LV+S)/HW (%)	Mean ± 2SD	Min.	Max.
All Macropods (48)	62.0	51.6–72.0	48.6	71.1
Kangaroos—Red, Western Grey (36)	62.0	52.2–72.4	49.1	71.1
Wallabies—YFRW, Tammar (12)	60.4	49.9–70.8	0.49	68.0
Koala (6)	59.5	46.5–72.5	51.7	69.5

**Table 9:** Cardiac measurements for macropods with cardiac disease (H = higher and L = lower than reference values found in normal macropods in this study).

Case	BW (kg)	HW (gm)	HW/BW (%)	LV+S (gm)	RV (gm)	LV (mm)	S (mm)	RV (mm)	(LV+S)/RV	LV:RV	LV:S	RV:S
1. Wallaroo	n/a	328	n/a	211	71	21	15	5	2.99	4.20 (H)	1.40	0.33
2. Matschie’s tree kangaroo	8.69	45.0	0.48	25.9	5.7	10	9.5	1.5	4.54 (H)	6.67 (H)	1.05	0.16 (L)

## Data Availability

Data is contained within the article or Supplementary Material.

## Supplementary Materials

The following supporting information can be downloaded at: https://www.mdpi.com/article/10.3390/ani15101397/s1.
